# The Application of Machine Learning in Predicting Mortality Risk in Patients With Severe Femoral Neck Fractures: Prediction Model Development Study

**DOI:** 10.2196/38226

**Published:** 2022-08-19

**Authors:** Lingxiao Xu, Jun Liu, Chunxia Han, Zisheng Ai

**Affiliations:** 1 Department of Medical Statistics Tongji University Shanghai China

**Keywords:** machine learning, femoral neck fracture, hospital mortality, hip, fracture, mortality, prediction, intensive care unit, ICU, decision-making, risk, assessment, prognosis

## Abstract

**Background:**

Femoral neck fracture (FNF) accounts for approximately 3.58% of all fractures in the entire body, exhibiting an increasing trend each year. According to a survey, in 1990, the total number of hip fractures in men and women worldwide was approximately 338,000 and 917,000, respectively. In China, FNFs account for 48.22% of hip fractures. Currently, many studies have been conducted on postdischarge mortality and mortality risk in patients with FNF. However, there have been no definitive studies on in-hospital mortality or its influencing factors in patients with severe FNF admitted to the intensive care unit.

**Objective:**

In this paper, 3 machine learning methods were used to construct a nosocomial death prediction model for patients admitted to intensive care units to assist clinicians in early clinical decision-making.

**Methods:**

A retrospective analysis was conducted using information of a patient with FNF from the Medical Information Mart for Intensive Care III. After balancing the data set using the Synthetic Minority Oversampling Technique algorithm, patients were randomly separated into a 70% training set and a 30% testing set for the development and validation, respectively, of the prediction model. Random forest, extreme gradient boosting, and backpropagation neural network prediction models were constructed with nosocomial death as the outcome. Model performance was assessed using the area under the receiver operating characteristic curve, accuracy, precision, sensitivity, and specificity. The predictive value of the models was verified in comparison to the traditional logistic model.

**Results:**

A total of 366 patients with FNFs were selected, including 48 cases (13.1%) of in-hospital death. Data from 636 patients were obtained by balancing the data set with the in-hospital death group to survival group as 1:1. The 3 machine learning models exhibited high predictive accuracy, and the area under the receiver operating characteristic curve of the random forest, extreme gradient boosting, and backpropagation neural network were 0.98, 0.97, and 0.95, respectively, all with higher predictive performance than the traditional logistic regression model. Ranking the importance of the feature variables, the top 10 feature variables that were meaningful for predicting the risk of in-hospital death of patients were the Simplified Acute Physiology Score II, lactate, creatinine, gender, vitamin D, calcium, creatine kinase, creatine kinase isoenzyme, white blood cell, and age.

**Conclusions:**

Death risk assessment models constructed using machine learning have positive significance for predicting the in-hospital mortality of patients with severe disease and provide a valid basis for reducing in-hospital mortality and improving patient prognosis.

## Introduction

Femoral neck fracture (FNF) accounts for approximately 3.58% of all fractures in the entire body [1], exhibiting an increasing trend each year. According to a survey, in 1990, the total number of hip fractures in men and women worldwide was approximately 338,000 and 917,000, respectively [2]. In China, FNFs account for 48.22% of hip fractures [3].

The Medical Information Mart for Intensive Care (MIMIC) III database is a publicly available database commonly used in clinical research [4], which contains medical data on approximately 60,000 patients in the intensive care unit (ICU) at Beth Israel Deaconess Medical Center from 2001 to 2012. The ICU database is more dimensional, dense, and valuable in the field of medicine than the general patient electronic medical record database [5]. The large amount of data recorded from these treatments and examinations is conducive to the close observation of ICU patients to detect physiological changes associated with deterioration and to provide more valuable data for clinical research [6].

Currently, many studies have been conducted on postdischarge mortality and mortality risk in patients with FNF [7-9]. Sheikh et al [8] used backward stepwise likelihood ratio Cox regression model to comprehensively analyze the causes of death in patients with FNF fracture 30 days after surgery, and found that age, admission hemoglobin, and history of myocardial infarction were important influencing factors to increase mortality. Dhingra et al [9] retrospectively analyzed the influencing factors of 1-year postoperative mortality in patients older than 60 years with FNF, and found that smoking, hypertension, diabetes, low hemoglobin, elevated white blood cell count, and surgical delay (>1 week) were significantly associated with higher 1-year postoperative mortality. Frost et al [7] used logistic regression model to determine the risk factors of postoperative nosocomial death in patients with FNF and used a nomogram model to predict the risk of death in a short period of time. Studies showed that age, gender, and complications were the main risk factors for nosocomial death in patients with femoral neck fracture. However, there have been no definitive studies on in-hospital mortality or its influencing factors in such patients with severe FNF admitted to the ICU. Therefore, in this study, we used the electronic case information of FNF patients recorded in the MIMIC database to examine the factors of in-hospital mortality in patients with FNF using a machine learning model to identify indicators that are meaningful for predicting in-hospital mortality and to provide preventive measures to reduce in-hospital mortality in patients as early as possible.

## Methods

### Data Source

Patient data from MIMIC-III were used for this study, which is a database commonly used in critical care big data studies; it contains clinical information such as demographics, vital signs, laboratory tests, treatment protocols, and diagnostic codes for 46,520 patients in ICU.

### Ethical Considerations

The MIMIC-III database was approved by the Massachusetts Institute of Technology (Cambridge, MA) and Beth Israel Deaconess Medical Center (Boston, MA). The authors have obtained the database download and use right through Protecting Human Research Participants Exam (No. 38335409). Therefore, the ethical approval statement and the need for informed consent were waived for this manuscript.

### Inclusion and Exclusion Criteria

**Figure 1:**
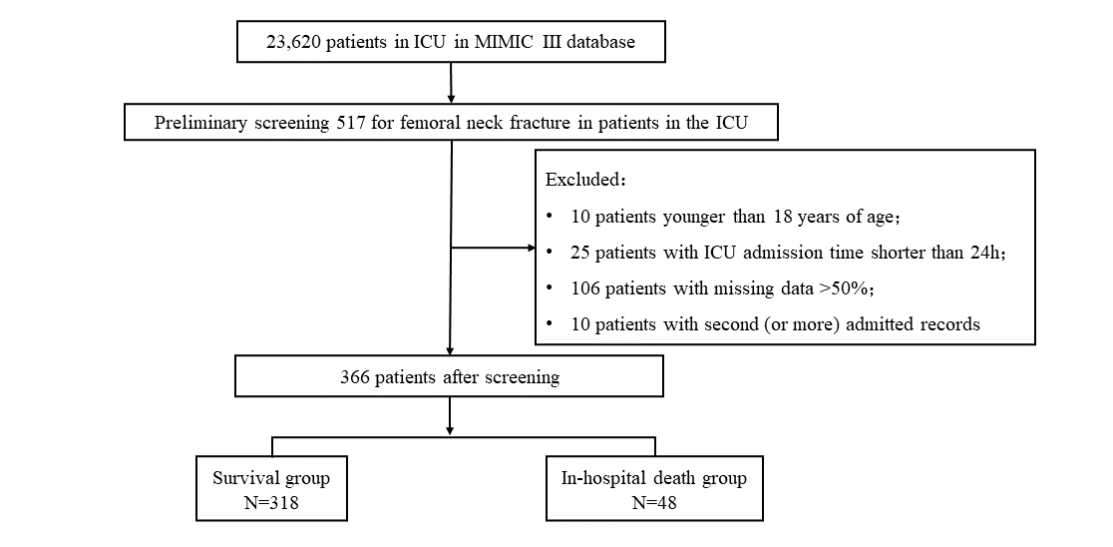
Case screening flowchart. ICU: intensive care unit; MIMIC: Medical Information Mart for Intensive Care.

In this study, patients admitted to the ICU for FNFs were extracted from the MIMIC-III database according to their diagnosis codes. The case information included in this study was based on the first admission, and data from patients with the first diagnosis code of FNF, including rotator fracture and intertrochanteric fracture, were selected according to the order of diagnosis codes. Patients aged ≤18 years or with ICU length of stay <24 hours were excluded, as were patients with grossly incomplete medical data records (>50% numbers missing). The case screening process is shown in Figure 1.

### Data Collection

Data were collected based on clinical experience, published literature, and data recorded in the MIMIC III database. Data collection for patients with FNFs was performed in the following 3 main areas: (1) demographic information—sex, age, BMI, length of ICU stay, history of previous illness, and Simplified Acute Physiology Score II (SAPS II); (2) physiological and biochemical indices within 24 hours after admission to the ICU—serum calcium, hemoglobin, hematocrit, lactate, cardiac troponin T level, creatine kinase (CK), creatine kinase isoenzyme (CKMB), vitamin D, red blood cells, white blood cells, and creatinine; and (3) outcome—whether in-hospital death occurred after admission to the ICU in patients with critical FNFs.

### Data Preprocessing

The variables included in the study were screened to exclude cases with more than 50% missing values. For cases with no more than 50% missing data, random forest (RF) algorithm was used to impute variables containing missing values sequentially in a loop [10]. The common methods for filling missing data are the mean, plurality, median, and fixed value methods, and the RF algorithm is a promising method for filling missing data. The missing values are used as new labels, and the model is built to obtain predicted values for filling. The RF algorithm for filling in missing data is capable of handling mixed types of missing data and has the potential to scale up to big data environments.

Since the outcome labels extracted in this study are unbalanced (48/366, 13.1% cases in the death group and 318/366, 86.9% cases in the survival group), the prediction results of the model trained by the machine learning algorithm are prone to bias for the unbalanced data set; therefore, the original data set needs to be balanced. In this study, the synthetic minority oversampling technique (SMOTE) function in the “imblearn” library of Python (Python Software Foundation) is used to achieve the balanced processing of the data set. The SMOTE algorithm is implemented by randomly selecting a sample y from their k-nearest neighbors for each sample x in a relatively small number of mortality sample sets, and randomly synthesizing a new mortality sample on the x, y line. A total of 48 samples from the original mortality group were analyzed, and then 270 new mortality samples were randomly synthesized and added to the data set to finally obtain a new balanced data set (mortality group: survival group = 1:1).

The linear function normalization method was used in this study to normalize the newly balanced data set. Commonly used methods are linear function normalization (min-max scaling) and 0-mean normalization (*z*-score standardization). The normalization process is used to eliminate the computational errors caused by different data levels and normalize the data to the range of 0-1 to ensure that each feature is treated equally by the classifier.

The normalized data set was randomly assigned to the test set and the training set at a ratio of 7:3. Finally, 445 cases were obtained for training the prediction model, and 191 cases were used to verify the predictive performance of the model.

### Model Construction

Currently, logistic regression is one of the commonly used methods for identifying risk factors that predict the occurrence of complications [11,12]. In an open calcaneal fracture study, compared to the traditional logistic regression model, machine learning methods have 30% higher accuracy and are more suitable for clinical applications [13].

RF is an integrated learning algorithm consisting of multiple decision trees formed by randomly adding back resampled samples, which is suitable for problems where the number of samples is much smaller than the number of features [14]. It also has the advantages of robust effect, fast learning speed, strong generalization ability, and good classification performance for missing data and imbalanced data [15].

Backpropagation neural network (BPNN) is a feed-forward, and the most widely used, neural network [16]. The algorithm has high self-learning and self-adaptive ability, strong generalization ability, and good prediction performance for untrained data. At the same time, the BPNN has high fault tolerance; that is, even if the system is damaged locally, it can still work normally [17].

Extreme gradient boosting (XGBoost) algorithm is a mainstream machine learning algorithm based on tree model boosting [18]. It continuously updates the error or residual of the model by adding tree models and then adjusts the weight of the misclassification results so that the model can select samples more intelligently and reduce the errors generated by the model. The XGBoost algorithm has been widely used in clinical studies for predicting the occurrence of diseases and predicting adverse patient outcomes and has been shown to be more effective than other machine learning models in several studies [19-21].

Therefore, in this study, 3 algorithms, namely RF, BPNN, and XGBoost, were used to construct machine learning prediction models (Multimedia Appendix 1).

### Statistical Analysis and Model Evaluation

The PostgreSQL database system was used to extract the data. Statistical analysis was performed using SPSS 22.0 (IBM Corp), and data cleaning, model construction, and performance evaluation were performed using Python 3.8. All continuous variables are expressed as medians (quartiles), and count data are expressed as the number of cases (percentages). The Mann-Whitney *U* test was used for univariate analysis of continuous variables, and Fisher exact test was used for univariate analysis of categorical variables. The Pearson ^2^ test was used for the analysis of variance of the machine learning model results. *P*<.05 was considered to be a statistically significant difference.

The model evaluation indices were the area under the receiver operating characteristic curve (AUROC), accuracy, precision, sensitivity, specificity, and *F*_1_-score.

## Results

### Basic Characteristics of Patients With Severe FNFs

A total of 366 eligible patients with FNF with a mean age of 78 (SD 20.4) years were screened. Compared with surviving patients, in-hospital death occurred in older patients with a mean age of 83 (SD 17.8) years (*P*<.05). The SAPS II score, lactate dehydrogenase level, and creatinine level of patients in the death group were all significantly higher than those in the surviving group (*P*<.05) (Table 1).

**Table 1:** Baseline data of patients in the intensive care unit (ICU) with a femoral neck fracture.

Characteristics	Patients included (n=366)	Survival patients (n=318)	Death patients (n=48)	*P* value
Male, n (%)	193 (52.7)	172 (54.1)	21 (43.8)	.18
Female, n (%)	173 (47.3)	146 (45.9)	27 (56.2)	.18
Diabetes, n (%)	67 (18.3)	60 (18.9)	7 (14.6)	.47
Hypertension, n (%)	149 (40.7)	130 (40.9)	19 (39.6)	.87
Coronary, n (%)	86 (23.5)	70 (22.0)	16 (33.3)	.09
LOS^a^ (h) in ICU (IQR)	2.7 (1.3-4.9)	2.6 (1.4-4.7)	3.0 (1.2-6.1)	.94
BMI (IQR)	25.1 (21.0-31.3)	25.6 (21.1-31.5)	23.9 (20.6-28.6)	.17
Age (years; IQR)	78.0 (58.0-87.0)	76.5 (57.0-86.0)	83.0 (74.5-90.0)	.002
SAPS II^b^ score (IQR)	39.0 (27.8-40.0)	36.0 (27.0-45.0)	52.0 (39.5-65.8)	<.001
Calcium (IQR)	1.092 (1.1-1.1)	1.092 (1.1-1.1)	1.094 (1.1-1.1)	.41
Hematocrit (IQR)	22.33 (22.1-22.6)	22.35 (22.1-22.6)	22.25 (22.0-25.1)	.41
Hemoglobin (IQR)	7.610 (7.5-7.9)	7.612 (7.5-7.9)	7.579 (7.5-8.4)	.38
Lactate (IQR)	2.127 (1.8-2.9)	2.095 (1.8-2.8)	2.678 (2.0-4.7)	.001
TnT^c^ (IQR)	0.040 (0.0-0.1)	0.041 (0.0-0.1)	0.038 (0.0-0.1)	.69
CK (IQR)	156.5 (64-584.3)	171.0 (63.7-601.3)	133.0 (77.4-445.5)	.60
CKMB (IQR)	5.000 (3.3-12.0)	5.000 (3.3-12.0)	4.925 (3.5-12.6)	.69
Vitamin D (IQR)	218.7 (191.1-246.5)	218.7 (191.6-246.0)	216.1 (189.4-252.7)	.73
Red blood cell (IQR)	3.435 (3.0-3.9)	3.425 (3.0-3.9)	3.470 (3.0-3.9)	.77
White blood cell (IQR)	10.30 (7.4-13.7)	10.25 (7.4-13.7)	11.01 (7.6-14.0)	.67
Creatinine (IQR)	0.90 (0.7-1.3)	0.90 (0.7-1.2)	1.25 (0.7-1.6)	.01

^a^LOS: length of stay.

^b^SAPS II: Simplified Acute Physiology Score II.

^c^TnT: troponin T.

### Ranking of the Importance of Characteristic Variables

**Figure 2:**
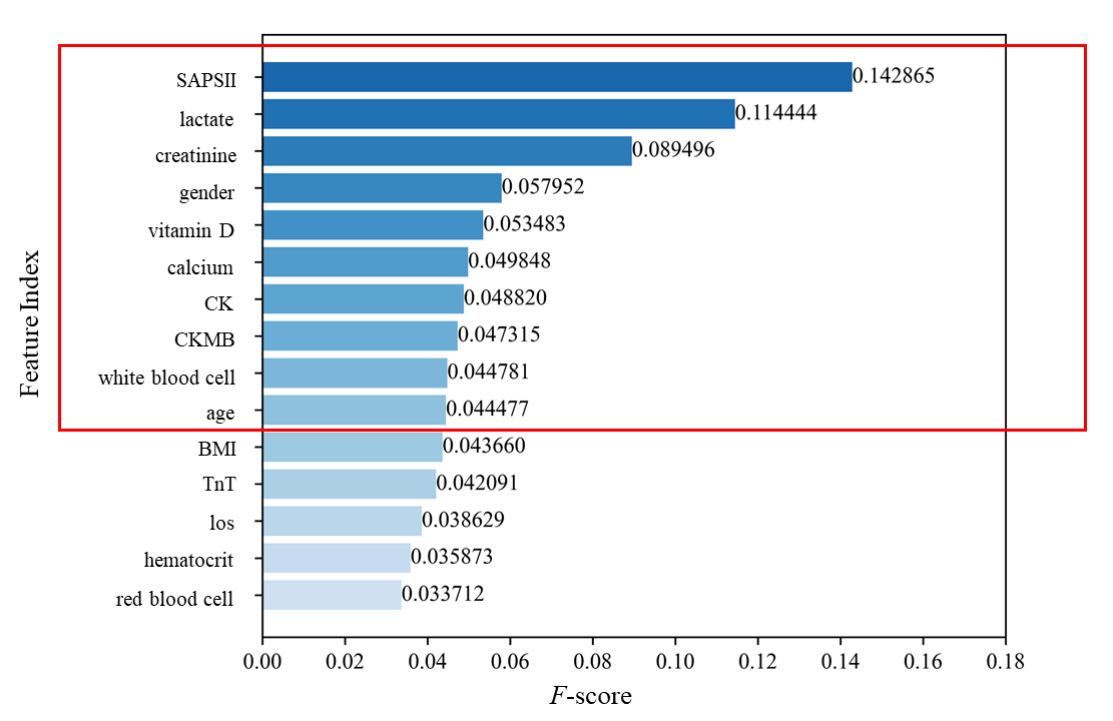
Ranking of important features in the model. CK: creatine kinase; CKMB: creatine kinase isoenzyme; los: length of stay; SAPII: Simplified Acute Physiology Score II; TnT: troponin T.

The RF model was used to rank the importance of characteristic variables, and the top 10 variables of characteristic importance (Figure 2) were SAPS II, lactate, creatinine, gender, vitamin D, calcium, CK, CKMB, white blood cell, and age. All biochemical indices were measured within 2 hours after admission to the ICU.

### Model Evaluation

#### Receiver Operating Characteristic Curve

**Figure 3:**
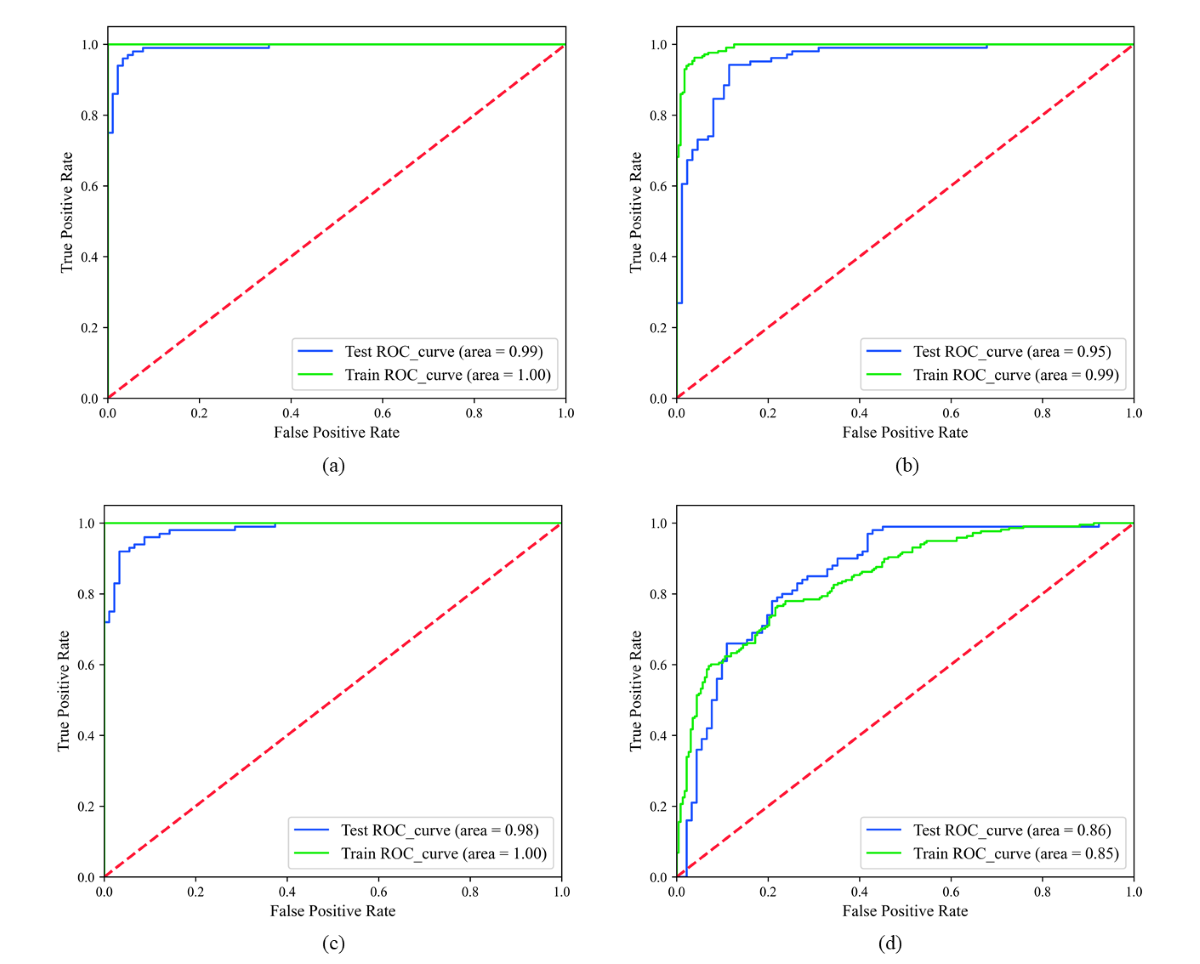
Receiver operating characteristic (ROC) curves of 4 prediction models: (a) random forest; (b) backpropagation neural network; (c) extreme gradient boosting; and (d) logistic regression.

Three machine learning models and a traditional logistic model were constructed on the training set and verified on the test set. The 3 machine learning models are RF, BPNN, and XGBoost. The receiver operating characteristic curves of the 4 prediction models were obtained, as shown in Figure 3. The AUROCs of the 4 models on the training set were 1.0, 0.99, 1.00, and 0.85, and the AUROCs on the test set were 0.99, 0.95, 0.98, and 0.86, respectively. Among them, the best results observed for the RF and XGBoost models, and the second-best for the BPNN, but the AUROCs of the machine learning models were all above 0.95. The prediction results of the 4 prediction models are analyzed for differences, and the results are shown in Table 2. The prediction accuracy of the three machine learning models on the test set is better than that of the traditional Logistic regression model, but the significant difference is not statistically significant (*P*>.05).

**Table 2:** Significance analysis of the prediction results of 4 models.

Prediction models	Outcome, n (%)		χ² (*df*)	*P* value
	In-hospital death	Survival		
RF^a^	103 (53.93)	88 (46.07)	2.240 (3)	.52
BPNN^b^	104 (54.45)	87 (45.55)	2.240 (3)	.52
XGBoost^c^	101 (52.88)	90 (47.12)	2.240 (3)	.52
Logistic regression	91 (47.64)	100 (52.36)	2.240 (3)	.52

^a^RF: random forest.

^b^BPNN: backpropagation neural network.

^c^XGBoost: extreme gradient boosting.

#### Confusion Matrix

**Figure 4:**
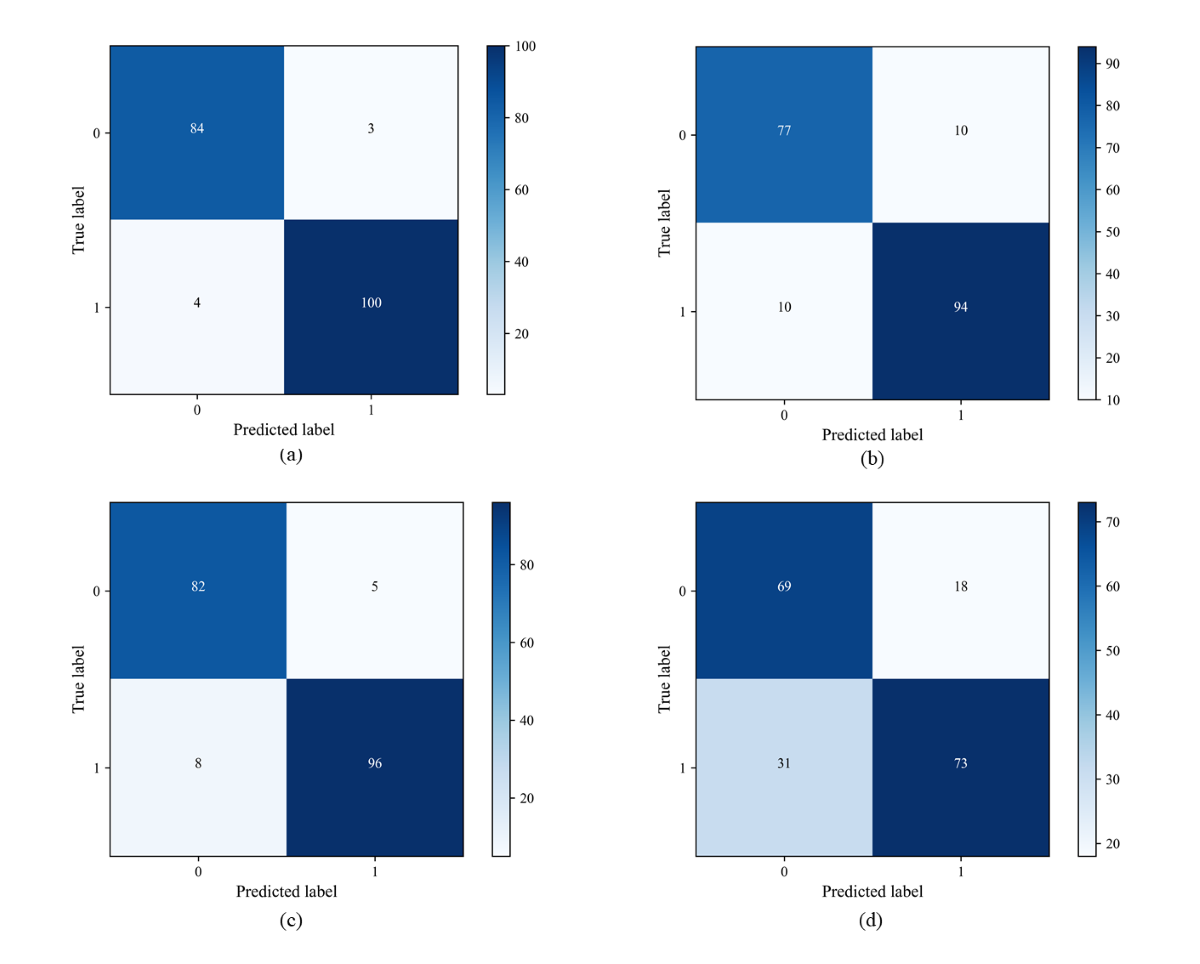
Confusion matrices for 4 prediction models; label 1 for the in-hospital death group and label 0 for the survival group: (a) random forest; (b) backpropagation neural network; (c) extreme gradient boosting; and (d) logistic regression.

The predictive performance of the 4 models was evaluated using accuracy, precision, sensitivity, specificity, and *F*_1_-score. The RF model had the best overall prediction with accuracy, precision, sensitivity, specificity, and *F*_1_-scores of 0.96, 0.97, 0.96, 0.97, and 0.92, respectively. The *F*_1_-score of both the XGBoost and BPNN was 0.89, but the accuracy, precision, sensitivity, and specificity of XGBoost were higher than those of the BPNN. All 3 machine learning models outperformed the traditional logistic regression model (Figure 4) in terms of prediction performance (Table 3).

**Table 3:** The prediction performance evaluation of four models.

Prediction model	AUROC^a^	Accuracy	Precision	Sensitivity	Specificity	*F*_1_-score
RF^b^	0.99	0.96	0.97	0.96	0.97	0.92
BPNN^c^	0.95	0.90	0.90	0.90	0.89	0.89
XGBoost^d^	0.98	0.93	0.95	0.92	0.94	0.89
Logistic regression	0.86	0.74	0.80	0.70	0.79	0.79

^a^AUROC: area under the receiving operating characteristic curve.

^b^RF: random forest.

^c^BPNN: backpropagation neural network.

^d^XGBoost: extreme gradient boosting.

## Discussion

### Principal Findings

In this study, 3 high-performing machine learning algorithms were selected to develop in-hospital mortality risk prediction models for patients with severe FNFs, including an RF model, a BPNN model, and an XGBoost model. The 3 machine learning models exhibited excellent performance on both the training and validation sets, with AUROC of the test set being 0.99, 0.95, and 0.98, respectively, and with better predictive performance compared to the traditional statistical logistic model. Meanwhile, the RF model was used in this study to rank the common predictors by calculating the importance of the feature variables. SAPS II, lactate, creatinine, gender, vitamin D, calcium, CK, CKMB, white blood cell, and age were further identified as significant predictors of death in patients with FNFs.

### Comparison With Prior Work

The logistic model, a traditional statistical prediction model, has been more widely used in the prediction of morbidity and mortality in FNF [22]. However, logistic regression is more sensitive to multiple covariance data; it is difficult to deal with the problem of data imbalance; the accuracy of the model is low; and the ability to fit the true distribution of the data is poor. In recent years, machine learning has been continuously applied to the prediction of disease occurrence and adverse outcomes in medicine. For example, the risk of acute kidney injury in patients in ICU was predicted using logistic regression, RF, and LightGBM algorithms by Gao [23]. The 3 models predicted the risk of acute kidney injury after 24 hours with increasing sensitivity, and the model efficacy of the RF and LightGBM algorithms was significantly better than that of logistic regression. Huan et al [24] used machine learning to construct models to predict and analyze the risk factors of femoral head necrosis after internal fixation in patients with FNF, and the results proved that there was a good consistency between the predicted probability of machine learning and the actual risk of necrosis. In this study, the prediction effect of machine learning models was compared with that of the traditional logistic regression model, and it was confirmed that machine learning models had good performance in predicting in-hospital mortality of patients with severe FNF, which was consistent with the above conclusion.

Meanwhile, the RF model was used in this study to rank the common predictors by calculating the importance of the feature variables. SAPS II, lactate, creatinine, gender, vitamin D, calcium, CK, CKMB, white blood cell, and age were further identified as significant predictors of death in patients with FNFs. In a previous study, Seitz et al [25] found that defective bone mineralization and a decrease in 25-hydroxy vitamin D were associated with increased mortality in FNFs. 25-hydroxy vitamin D is the primary form of vitamin D present in the blood. Vitamin D and serum calcium were important, influential factors affecting in-hospital mortality in patients with FNFs in this study, which validated this finding, suggesting that balancing serum 25-hydroxy vitamin D levels through calcium supplementation and other measures in clinical treatment may reduce mortality in FNFs. In a prospective controlled study by Paccou et al [26], lactate dehydrogenase levels and creatinine levels were significant predictors of bone mineral density (BMD) loss; this is while BMD was associated with mortality, and faster BMD loss was associated with a higher risk of death [27], which is consistent with the results of this study. In addition, compared to previous studies regarding the prediction of mortality in FNFs [28-30], this study found that the SAPS II score was also significant for predicting mortality in patients. SAPS II consists of 12 physiological variables, age, type of hospitalization, and 3 types of chronic disease, and the measurement of SAPS II daily after admission to the ICU can predict the risk of death [31]. However, in existing prediction studies [32-34], the SAPS II score is commonly used in prognostic studies of patients with neurological diseases, abdominal infections, and respiratory distress, though there are fewer studies on the predictive ability of the SAPS II critical score in FNFs. The results of this study are important for further refining the prediction of morbidity and mortality in patients with FNFs.

### Limitations

This study also has some limitations. First, this was a single-center study based on the MIMIC III database without external database validation, and the performance of the model needs to be further validated by prospective studies. Second, the interpretability of the machine learning model was poor, and although feature importance ranking was performed, the causal relationship between these features and in-hospital mortality in patients with FNFs could not be evaluated from a statistical perspective. Finally, some imaging metrics could not be included in the model due to limitations in the available data types in the MIMIC III database. Next, we will further integrate the existing model with the domestic database to validate the model performance, adjust the parameters to improve the model performance, and better adapt the model to the domestic database. Furthermore, we will extend the study timeline to establish a clinically applicable in-hospital mortality risk prediction model for patients with severe FNFs.

### Conclusions

In summary, we used patients’ clinical data to develop 3 machine learning models for predicting the risk of in-hospital death in patients with severe FNFs. The prediction performance of all 3 machine learning models was better than that of the traditional logistic model, and the RF model displayed the best prediction performance among the 3 models. In the future, after validating the domestic database and adjusting the model parameters, this model can be applied to clinical practice to better assist clinicians in decision-making, adjust treatment plans for patients with severe FNFs, better allocate medical supplies, and reduce the occurrence of adverse outcomes. Considering that MIMIC is a foreign database with fewer Asian patients, which is not universal for domestic FNF cases, more domestic patient data will be included in future work to adjust the model to make it more compatible with the characteristics of the domestic FNF population.

## Appendix

Multimedia Appendix 1 Paper code.

## Abbreviations

AUROC: area under the receiving operating characteristic curve
BMD: bone mineral density
BPNN: backpropagation neural network
CK: creatine kinase
CKMB: creatine kinase isoenzyme
FNF: femoral neck fracture
ICU: intensive care unit
MIMIC: Medical Information Mart for Intensive Care
RF: random forest
SAPS II: Simplified Acute Physiology Score II
SMOTE: synthetic minority oversampling technique
XGBoost: extreme gradient boosting
