# Supplementary material for: The Application of Machine Learning in Predicting Mortality Risk in Patients With Severe Femoral Neck Fractures: Prediction Model Development Study
Source: JMIR Bioinform Biotechnol. 2022 Aug 19;3(1):e38226. doi: 10.2196/38226 (PMC11135225; doi:10.2196/38226)
Supplement: Multimedia Appendix 1 [file bioinform_v3i1e38226_app1.pdf]

```
# 导入库
import pandas as pd
import numpy as np
import pandas as pd
import matplotlib.pyplot as plt
from sklearn.datasets import load_boston
from sklearn.impute import SimpleImputer
from sklearn.ensemble import RandomForestRegressor
from sklearn.model_selection import cross_val_score
import xgboost as xgb
# 导入数据
original_data0 = pd.read_excel(io='C:/Users/10699/Desktop/xlx/data', sheet_name="Sheet1",
header=0)
original_data=np.array(original_data0)[:,3:]
# vari_name = list(original_data)
mystring_data=(np.array(original_data))[:,1].reshape(-1,1)
myfloat_data=np.hstack((np.array(original_data)[:,0].reshape(-1,1),np.array(original_data)[:,2:-2]))
myfloat_data[myfloat_data=='NotDone']=np.nan
myfloat_data1=np.array(myfloat_data[:,:],dtype=np.float)[:,:]

#判断有缺失的列
ifloss=np.zeros((366,20)) #生成 366*20 的全 0 矩阵 ifloss
for j in range(len(myfloat_data1[0,:])):      #j 从 0 循环到 12(列的长度)
    for i in range(len(myfloat_data1)):       #i 从 0 循环到 366(行的长度)
        if np.isnan(myfloat_data1[i,j]):       #判断第 i 行第 j 列的数是否是 nan
            ifloss[i,j]=1                        #是 nan 的话 ifloss 矩阵对应位置变成
1
# lossLineNum=sum(sum(ifloss)!=0)     #lossLineNum 是有缺失数据的列数
# lossLineIndex=np.array(np.nonzero(sum(ifloss))).reshape(6)

#得到有缺失数据的列索引(由少到多)
sortindex=list((np.argsort(sum(ifloss))))
for i in range(12):
    if sum(ifloss)[i]==0:
        sortindex.remove(i)

# data = pd.DataFrame(myfloat_data1)
# filldata = data.iloc[:,5]
# data=data.iloc[:,data.columns != 5]
#data_0
=SimpleImputer(missing_values=np.nan,strategy='constant',fill_value=0).fit_transform(data)71qd
vb

# Ytrain = filldata[filldata.notnull()]
```

```
# Ytest = filldata[filldata.isnull()]
# Xtrain = data_0[Ytrain.index,:]
# Xtest = data_0[Ytest.index,:]

# rfc = RandomForestRegressor(n_estimators=100)
# rfc = rfc.fit(Xtrain, Ytrain)
# Ypredict = rfc.predict(Xtest)

# X_missing_reg.loc[X_missing_reg.iloc[:,6].isnull(),6] = Ypredict
# X_missing_reg.isnull().sum()

myfloat_data2=myfloat_data1.copy()
# 循环插值
for i in sortindex:
    data = pd.DataFrame(myfloat_data2)     # DataFrame 格式可以使用很多函数
    filldata = data.iloc[:,i]                    # 将需要填充的数据单独列出
    data=data.iloc[:,data.columns != i]
    data_0
=SimpleImputer(missing_values=np.nan,strategy='constant',fill_value=0).fit_transform(data)
    Ytrain = filldata[filldata.notnull()]
    Ytest = filldata[filldata.isnull()]
    Xtrain = data_0[Ytrain.index,:]
    Xtest = data_0[Ytest.index,:]
    rfc = RandomForestRegressor(n_estimators=100)   # 种子数越大越好
    rfc = rfc.fit(Xtrain, Ytrain)
    Ypredict = rfc.predict(Xtest)
    myfloat_data2[ifloss[:,i]==1,i] = Ypredict

# 合并数据
mystring_data[mystring_data=='F'] = 0
mystring_data[mystring_data=='M'] = 1
mystring_data[mystring_data=='carevue'] = 0
mystring_data[mystring_data=='metavision'] = 1
mystring_data[mystring_data=='both'] = 2
mystring_data1 = np.array(mystring_data,dtype=np.float)
total_data=np.hstack((mystring_data1,myfloat_data2))

# 填补后数据
original_data = pd.read_excel(io='C:/Users/10699/Desktop/imputedata.xlsx',
sheet_name="after",header=0)
mystring_data=(np.array(original_data))[:,0:2]
myfloat_data=np.array(original_data)[:,2:-1]
myfloat_data1=np.array(myfloat_data,dtype=np.float)[:,:]
mystring_data[mystring_data=='F'] = 0
```

```
mystring_data[mystring_data=='M'] = 1
mystring_data[mystring_data=='carevue'] = 0
mystring_data[mystring_data=='metavision'] = 1
mystring_data[mystring_data=='both'] = 2
mystring_data1 = np.array(mystring_data,dtype=np.float)
total_data=np.hstack((mystring_data1,myfloat_data1))

original_data = pd.read_excel(io='C:/Users/10699/Desktop/8.4.xlsx', sheet_name="填补后数据",header=0)
total_data=(np.array(original_data))

################ 导入数据 ################################################
import pandas as pd
import numpy as np
original_data0 = pd.read_excel(io='C:/Users/10333/Desktop/8.4.xlsx', sheet_name="均衡数据", header=0)
total_data = np.array(original_data0)

# total_data_1=total_data0[total_data0[:,-1]==1]
# from imblearn.over_sampling import SMOTE
# X_resampled_smote, y_resampled_smote = SMOTE().fit_resample(total_data0[:,:-1], total_data0[:,-1])
# total_data=np.hstack((X_resampled_smote,y_resampled_smote.reshape(-1,1)))
# out_data = np.array(sorted(total_data, key = lambda x:x[-1]))
# 导入标签
Y_data = np.array(total_data)[:,-1]
# Y_data1 = np.array(Y_data,dtype=np.float)
# total_data1 = np.column_stack((total_data,Y_data1))

#1 随机划分训练集和验证集
train_index=np.random.choice(range(len(original_data0)),
int(len(original_data0)*0.7),replace=False)
train_index.sort()
test_index = list(set(range(636))-set(train_index))
test_index = np.array(test_index)

# 归一化
from sklearn import preprocessing
from sklearn.preprocessing import MinMaxScaler
min_max_scaler = preprocessing.MinMaxScaler()
data=min_max_scaler.fit_transform(total_data)
data_train = data[train_index].copy()
data_test = data[test_index].copy()
data_train_in = data_train[:,:-1]
```

```
data_train_out = data_train[:,-1].reshape(-1,1)
data_test_in = data_test[:,:-1]
data_test_out = data_test[:,-1].reshape(-1,1)


# 导入库
import pandas as pd
import numpy as np
import pandas as pd
import matplotlib.pyplot as plt
from sklearn.datasets import load_boston
from sklearn.impute import SimpleImputer
from sklearn.ensemble import RandomForestRegressor
from sklearn.model_selection import cross_val_score

################ 随机森林 #####################################
from sklearn.model_selection import KFold, cross_val_score as CVS, train_test_split as TTS
# Xtrain,Xtest,Ytrain,Ytest = TTS(data_train_in,data_train_out,test_size=0.3,random_state=420)
data_test_pre=data_test_out.copy()
from sklearn.ensemble import RandomForestClassifier

from sklearn import metrics

Classifier_RF = RandomForestClassifier(n_estimators=700)   # 种子数越大越好
Classifier_RF.fit(data_train_in, data_train_out)
data_test_pre_rf = Classifier_RF.predict_proba(data_test_in)
maxindex_rf = np.argmax(data_test_pre_rf,axis=1)
test_y_score_rf = data_test_pre_rf[:,1]
data_train_pre_rf = Classifier_RF.predict_proba(data_train_in)
train_y_score_rf = data_train_pre_rf[:,1]
accuarcy_score_rfc = Classifier_RF.score(data_test_in, maxindex_rf)



#画图 plt.scatter(data_test_out,data_test_pre,s=10)
plt.xlabel('theoretical value',size=13)
plt.ylabel('predict value',size=13)
# calc the trendline
z = np.polyfit(data_test_out.reshape(-1), data_test_pre.reshape(-1), 1)
p = np.poly1d(z)
plt.plot(np.linspace(0.2,0.9,10),p(np.linspace(0.2,0.9,10)),"r--")


# fit = model.fit(x_train, y_training)
```

```
# # ROC
# y_score = model.fit(x_train, y_training).predict_proba(x_test)   #  随机森林
# fpr, tpr, thresholds = roc_curve(y_test, y_score[:, 1])
# roc_auc = auc(fpr, tpr)


mycolor1=[[255/255,28/255,58/255],[17/255,238/255,0/255],[30/255,84/255,1]]
mycolor2=[[89/255,205/255,198/255],[200/255,45/255,168/255]]


import matplotlib as mpl
import matplotlib.pyplot as plt

#  设置西文字体为新罗马字体
from matplotlib import rcParams
config = {
    "font.family":'Times New Roman',   #  设置字体类型
    "font.size": 10,
#        "mathtext.fontset":'stix',
}
rcParams.update(config)
plt.title("Times New Roman", )
plt.axis('off')
plt.show()

cmap=plt.get_cmap('Blues')
newcolors=cmap(np.linspace(0, 1, 25))
#####################################特征变量重要性排序#######
import_level = Classifier_RF.feature_importances_ #这个方法可以调取关于特征重要程度

x=['SAPSII','lactate','creatinine','gender','Vitamin
D','calcium','CK','CKMB','whitebloodcell','age','BMI','TnT','los','hematocrit','redbloodcell']
y=[0.142865,0.114444,0.0894964,0.0579521,0.053483,0.0498482,0.0488198,0.047315,0.044780
6,0.044477,0.0436601,0.0420915,0.0386291,0.0358734,0.0337116]
X,Y=x.copy(),y.copy()
for i in range(len(x)):
    X[i]=x[len(x)-i-1]
    Y[i]=y[len(y)-i-1]
plt.figure(figsize=(6,5))
# b = plt.barh(X[:5],Y[:5],color='gray')
a = plt.barh(X[:],Y[:],color=newcolors[5:20])
plt.xlabel('F-Score',fontsize=12)
plt.ylabel('Feature Index',fontsize=12)
plt.xlim([0, 0.18])
```

```
for rect in a:
        w = rect.get_width()
        plt.text(w, rect.get_y()+rect.get_height()/2, '%f' %
                    (w), ha='left', va='center')
plt.savefig('C:/Users/10333/Desktop/feature_importance',dpi=600,bbox_inches = 'tight')

plt.savefig('C:/Users/10333/Desktop/Rf_feature_importance',dpi=600,bbox_inches = 'tight')
plt.show()


# #评价指标:AUC
# myTP=0;myTN=0;FP=0;FN=0
# from sklearn.metrics import roc_curve,auc,confusion_matrix
# # train_y_score_rf=rfc.predict_proba(data_train_in)
# # test_y_score_rf=rfc.predict_proba(data_test_in)
#    myFPR_train_rf,myTPR_train_rf,myTHRESHOLDS_train_rf=    roc_curve(data_train_out,
train_y_score_rf)
#      myFPR_test_rf,myTPR_test_rf,myTHRESHOLDS_test_rf=      roc_curve(data_test_out,
test_y_score_rf)
# myAUC_train_rf = auc(myFPR_train_rf,myTPR_train_rf)
# myAUC_test_rf = auc(myFPR_test_rf,myTPR_test_rf)
# import matplotlib.pyplot as plt
# lw = 2
# plt.figure(figsize=(6,5))
# plt.plot(myFPR_test_rf,myTPR_test_rf,c=mycolor1[2],label='Test ROC_curve (area = %0.2f)' %
myAUC_test_rf)
#    plt.plot(myFPR_train_rf,myTPR_train_rf,c=mycolor1[1],label='Train    ROC_curve    (area
= %0.2f)' % myAUC_train_rf)
# plt.plot([0, 1], [0, 1], color=mycolor1[0], lw=lw, linestyle='--')
# plt.xlabel('False Positive Rate',size=12)
# plt.ylabel('True Positive Rate',size=12)
# plt.xlim([0, 1.0])
# plt.ylim([0, 1.05])
# plt.legend(loc='lower right',fontsize=12)
#
plt.savefig('C:/Users/10699/Desktop/xlx/pic/20220220/RF_ROC_curve',dpi=600,bbox_inches='ti
ght')




#绘制混淆矩阵

def plot_confusion_matrix(cm, classes,normalize=False,cmap=plt.cm.Blues):
```

```
    """
    This function prints and plots the confusion matrix.
    Normalization can be applied by setting `normalize=True`.
    """
    if normalize:
        cm = cm.astype('float') / cm.sum(axis=1)[:, np.newaxis]
        print("Normalized confusion matrix")
    else:
        print('Confusion matrix, without normalization')
    print(cm)
    plt.imshow(cm, interpolation='nearest', cmap=cmap)
    plt.colorbar()
    tick_marks = np.arange(len(classes))
    plt.xticks(tick_marks, classes)
    plt.yticks(tick_marks, classes)
    fmt = '.2f' if normalize else 'd'
    thresh = cm.max() / 2.
    for i, j in itertools.product(range(cm.shape[0]), range(cm.shape[1])):
        plt.text(j, i, format(cm[i, j], fmt),
                 horizontalalignment="center",
                 color="white" if cm[i, j] > thresh else "black")
    plt.tight_layout()
    plt.ylabel('True label',fontsize=12)
    plt.xlabel('Predicted label',fontsize=12)

import itertools
cnf_matrix_rf = confusion_matrix(data_test_out, maxindex_rf) #计算混淆矩阵
class_names = [0,1]
plt.figure()
plot_confusion_matrix(cnf_matrix_rf, classes = class_names)    #绘制混淆矩阵
np.set_printoptions(precision=2)
rf_Accary=[(cnf_matrix_rf[1,1]+cnf_matrix_rf[0,0])/(cnf_matrix_rf[1,1]+cnf_matrix_rf[0,1]+cnf_matrix_rf[0,0]+cnf_matrix_rf[1,0])]
rf_Recall=[ cnf_matrix_rf[1,1]/(cnf_matrix_rf[1,1]+cnf_matrix_rf[1,0])]
rf_Precision=[cnf_matrix_rf[1,1]/(cnf_matrix_rf[1,1]+cnf_matrix_rf[0,1])]
rf_Specificity=[cnf_matrix_rf[0,0]/(cnf_matrix_rf[0,1]+cnf_matrix_rf[0,0])]
plt.savefig('C:/Users/10699/Desktop/xlx/pic/20220220/RF_Confusion_matrix',dpi=600,bbox_inches = 'tight')

##########################################################################


######### 建立 BP 神经网络模型 ######################################
import keras
```

```
from keras.optimizers import SGD,adam
from keras.utils import plot_model #画模型
from keras.models import Sequential
from keras.layers.core import Dense, Activation,Dropout
from keras.models import load_model
from keras.callbacks import LambdaCallback

# model = Sequential()   #层次模型
# model.add(Dense(16,input_dim=21,init='uniform')) #输入层，Dense 表示 BP 层
# model.add(Activation('sigmoid'))   #添加激活函数
# model.add(Dense(1,input_dim=16))   #输出层
# model.add(Activation('sigmoid'))   #添加激活函数
# model.compile(loss='binary_crossentropy', optimizer='adam',metrics=['accuracy']) #编译模型
# model.summary()
# plot_model(model, show_layer_names=0)
# myhistory=model.fit(data_train_in, data_train_out, nb_epoch = 500, batch_size = 6) #训练模型
100 次
# his=myhistory.history

Classifier_BP = Sequential()
Classifier_BP.add(Dense(64,input_dim=20,init='uniform')) #输入层，Dense 表示 BP 层
Classifier_BP.add(Activation('sigmoid'))   #添加激活函数
Classifier_BP.add(Dropout(0.1))
Classifier_BP.add(Dense(32,input_dim=64)) #输入层，Dense 表示 BP 层
Classifier_BP.add(Activation('sigmoid'))   #添加激活函数
Classifier_BP.add(Dropout(0.1))
Classifier_BP.add(Dense(16,input_dim=32))   #输出层
Classifier_BP.add(Activation('sigmoid'))   #添加激活函数
Classifier_BP.add(Dropout(0.1))
Classifier_BP.add(Dense(1,input_dim=16))   #输出层
Classifier_BP.add(Activation('sigmoid'))   #添加激活函数
Classifier_BP.compile(loss='binary_crossentropy', optimizer='adam',metrics=['accuracy']) #编译
模型
Classifier_BP.summary()
plot_model(Classifier_BP, show_layer_names=0)
test_accuracy_bp=[]
# caculate the acc after every epoch.
test_acc_callback = LambdaCallback(
    on_epoch_end=lambda epoch, logs:
        {
        test_accuracy_bp.append([Classifier_BP.evaluate(data_train_in,
data_train_out)[1],Classifier_BP.evaluate(data_test_in, data_test_out)[1]]),
        print([Classifier_BP.evaluate(data_train_in,
data_train_out)[1],Classifier_BP.evaluate(data_test_in, data_test_out)[1]])}
```

```
                              )
myhistory=Classifier_BP.fit(data_train_in, data_train_out, nb_epoch = 500, batch_size = 1,callbacks=[test_acc_callback]) #训练模型 100 次
his=myhistory.history
train_loss, train_accuracy = Classifier_BP.evaluate(data_train_in, data_train_out)
test_loss, test_accuracy = Classifier_BP.evaluate(data_test_in, data_test_out)
test_accuracy_bp=np.array(test_accuracy_bp)
Classifier_BP.save(r"C:\Users\10699\Desktop\xlx\BPmodel.hdf5")
# del Classifier_BP
# # 再次载入模型
# Classifier_BP = load_model(r"C:\Users\10699\Desktop\xlx\BPmodel.hdf5")

# # 画图
# import matplotlib.pyplot as plt
# plt.plot(np.linspace(1,500,500),test_accuracy[:,1],c=[89/255,205/255,198/255],label='loss')
# plt.plot(np.linspace(1,500,500),his['loss'],c=[89/255,205/255,198/255],label='loss')
# # plt.scatter(np.linspace(0,99,100),his['loss'],color=[89/255,205/255,198/255],marker='*',s=100)
# plt.plot(np.linspace(1,500,500),his['accuracy'],c=[200/255,45/255,168/255],label='accuracy')
# plt.xlabel('epoch',size=15)
# plt.ylabel('loss',size=15)
# plt.legend()
# plt.title('curve of loss & acc')
# # plt.xlim([0,150])
# # plt.ylim([0,2])

#4 预测，并还原结果
# modelfile = 'G:\pymodel\modelweight.h5' #神经网络保存
# model.save(modelfile) #保存模型
# model1=load_model('G:\pymodel\modelweight.h5')#加载模型
data_test_pre=Classifier_BP.predict(data_test_in)
data_test_pre[data_test_pre < 0.5]=0
data_test_pre[data_test_pre > 0.5]=1
err=data_test_pre-data_test_out

#评价指标
plt.figure(figsize=(6,5))
myTP=0;myTN=0;FP=0;FN=0
from sklearn.metrics import roc_curve,auc
train_y_true=data_train_out
train_y_score=Classifier_BP.predict(data_train_in)
test_y_true=data_test_out
test_y_score=Classifier_BP.predict(data_test_in)
myFPR_train_bp,myTPR_train_bp,myTHRESHOLDS_train_bp= roc_curve(train_y_true, train_y_score, pos_label=None, sample_weight=None, drop_intermediate=True)
```

```
myFPR_test_bp,myTPR_test_bp,myTHRESHOLDS_test_bp= roc_curve(test_y_true, test_y_score, pos_label=None, sample_weight=None, drop_intermediate=True)
myAUC_train_bp = auc(myFPR_train_bp,myTPR_train_bp)
myAUC_test_bp = auc(myFPR_test_bp,myTPR_test_bp)
lw = 2
plt.plot(myFPR_test_bp,myTPR_test_bp,c=mycolor1[2],label='Test ROC_curve (area = %0.2f)' % myAUC_test_bp)
plt.plot(myFPR_train_bp,myTPR_train_bp,c=mycolor1[1],label='Train ROC_curve (area = %0.2f)' % myAUC_train_bp)
plt.plot([0, 1], [0, 1], color=mycolor1[0], lw=lw, linestyle='--')
plt.xlabel('False Positive Rate',size=12)
plt.ylabel('True Positive Rate',size=12)
plt.xlim([0.0, 1.0])
plt.ylim([0.0, 1.05])
plt.legend(loc='lower right',fontsize=12)
plt.savefig('C:/Users/10699/Desktop/xlx/pic/20220220/BP_ROC_curve',dpi=600,bbox_inches = 'tight')
# import matplotlib.pyplot as plt
# plt.plot(myFPR_test,myTPR_test,c='b',label='test ROC_curve')
# plt.plot(myFPR_train,myTPR_train,c='r',label='train ROC_curve')
# plt.xlabel('FPR',size=12)
# plt.ylabel('TPR',size=12)
# plt.legend()
# plt.title('BP: curve of ROC')


#绘制混淆矩阵

def plot_confusion_matrix(cm, classes,normalize=False,cmap=plt.cm.Blues):
    """
    This function prints and plots the confusion matrix.
    Normalization can be applied by setting `normalize=True`.
    """
    if normalize:
        cm = cm.astype('float') / cm.sum(axis=1)[:, np.newaxis]
        print("Normalized confusion matrix")
    else:
        print('Confusion matrix, without normalization')
    print(cm)
    plt.imshow(cm, interpolation='nearest', cmap=cmap)
    plt.colorbar()
    tick_marks = np.arange(len(classes))
    plt.xticks(tick_marks, classes)
    plt.yticks(tick_marks, classes)
```

```
    fmt = '.2f' if normalize else 'd'
    thresh = cm.max() / 2.
    for i, j in itertools.product(range(cm.shape[0]), range(cm.shape[1])):
        plt.text(j, i, format(cm[i, j], fmt),
                 horizontalalignment="center",
                 color="white" if cm[i, j] > thresh else "black")
    plt.tight_layout()
    plt.ylabel('True label',fontsize=12)
    plt.xlabel('Predicted label',fontsize=12)

import itertools
cnf_matrix_bp = confusion_matrix(data_test_out, data_test_pre) #计算混淆矩阵
class_names = [0,1]
plt.figure()
plot_confusion_matrix(cnf_matrix_bp, classes = class_names)   #绘制混淆矩阵
np.set_printoptions(precision=2)
bp_Accary=[(cnf_matrix_bp[1,1]+cnf_matrix_bp[0,0])/(cnf_matrix_bp[1,1]+cnf_matrix_bp[0,1]+
cnf_matrix_bp[0,0]+cnf_matrix_bp[1,0])]
bp_Recall= [cnf_matrix_bp[1,1]/(cnf_matrix_bp[1,1]+cnf_matrix_bp[1,0])]
bp_Precision=[cnf_matrix_bp[1,1]/(cnf_matrix_bp[1,1]+cnf_matrix_bp[0,1])]
bp_Specificity=[cnf_matrix_bp[0,0]/(cnf_matrix_bp[0,1]+cnf_matrix_bp[0,0])]
plt.savefig('C:/Users/10699/Desktop/xlx/pic/20220220/BP_Confusion_matrix',dpi=600,bbox_inch
es = 'tight')

###################### 建立 XGBoost 模型 ##############################
from sklearn.model_selection import KFold, cross_val_score as CVS, train_test_split as TTS
from sklearn.metrics import accuracy_score, confusion_matrix, mean_squared_error,roc_auc_score
import pandas as pd
import numpy as np
import xgboost as xgb
from xgboost import plot_importance
import matplotlib.pyplot    as plt
from sklearn.model_selection import train_test_split
from sklearn.metrics import accuracy_score    #  准确率
from sklearn.model_selection import KFold, cross_val_score as CVS, train_test_split as TTS
from sklearn.metrics import accuracy_score, confusion_matrix, mean_squared_error,roc_auc_score
import pandas as pd
import numpy as np
import xgboost as xgb
from xgboost import plot_importance
import matplotlib.pyplot    as plt
from sklearn.model_selection import train_test_split
from sklearn.metrics import accuracy_score    #  准确率
from sklearn.model_selection import cross_val_score
```

```
from sklearn.model_selection import cross_validate
from sklearn.model_selection import KFold
from sklearn.model_selection import validation_curve
from sklearn.metrics import r2_score,mean_absolute_error

data_test_pre_xg=[]
xgtrain = xgb.DMatrix(data_train_in, label=data_train_out)
n_estimators = 1000
params = {'n_estimators':n_estimators, 'booster':'gbtree', 'max_depth':6, 'learning_rate':0.05,
                    'objective':'binary:logistic', 'subsample':1, 'colsample_bytree':1}
Classifier_XGB = xgb.XGBClassifier(**params)
params_xgb = params.copy() # 修改参数
num_round = params_xgb['n_estimators']
params_xgb['eta'] = params['learning_rate']
del params_xgb['n_estimators']
del params_xgb['learning_rate']
cv = KFold(n_splits=10, shuffle=True, random_state=100)
# xgboost 原生接口 进行交叉验证
res = xgb.cv(params_xgb, xgtrain, num_round, folds=cv, metrics='auc',early_stopping_rounds=50)
Classifier_XGB.set_params(n_estimators=res.shape[0])#把 clf 的参数设置成最好的树对应的参
数
Classifier_XGB.fit(data_train_in, data_train_out, eval_metric='auc')#训练 clf
data_test_pre_xg=Classifier_XGB.predict_proba(data_test_in)
test_y_score_xg = data_test_pre_xg[:,1]
maxindex_xg = np.argmax(data_test_pre_xg,axis=1)
data_train_pre_xg = Classifier_XGB.predict_proba(data_train_in)
train_y_score_xg = data_train_pre_xg[:,1]
data_test_pre_xg_class=0*data_test_pre_xg
for i in range(len(data_test_pre_xg_class)):
    data_test_pre_xg_class[i,int(maxindex_xg[i])]=1

accuarcy_score_xg = Classifier_XGB.score(data_test_in, maxindex_xg)

# 显示重要特征
#定义函数来显示柱状上的数值

cmap=plt.get_cmap('Blues')
newcolors=cmap(np.linspace(0, 1, 25))


x=['F8','F6','F19','F12','F1','F9','F18','F4','F14','F2','F6','F7','F0','F3','F17']
y=[249,237,234,227,194,182,177,176,164,163,143,126,126,125,117]
X,Y=x.copy(),y.copy()
for i in range(len(x)):
```

```
        X[i]=x[len(x)-i-1]
        Y[i]=y[len(y)-i-1]
plt.figure(figsize=(6,5))
# b = plt.barh(X[:5],Y[:5],color='gray')
a = plt.barh(X[:],Y[:],color=newcolors[5:20])
plt.xlabel('F-Score',fontsize=12)
plt.ylabel('Feature Index',fontsize=12)
plt.xlim([0, 300])
for rect in a:
        w = rect.get_width()
        plt.text(w, rect.get_y()+rect.get_height()/2, '%d' %
                        int(w), ha='left', va='center')
plt.savefig('C:/Users/10699/Desktop/xlx/pic/20220220/feature_importance',dpi=600,bbox_inches
= 'tight')



a = plot_importance(Classifier_XGB,height=0.5,max_num_features=15,grid=False,color='Blue')

plt.savefig('C:/Users/10699/Desktop/xlx/pic/20220220/xgb_feature_importance',dpi=600,bbox_in
ches = 'tight')
plt.show()


#评价指标:AUC
plt.figure(figsize=(6,5))
myTP=0;myTN=0;FP=0;FN=0
from sklearn.metrics import roc_curve,auc,confusion_matrix
# train_y_score_rf=rfc.predict_proba(data_train_in)
# test_y_score_rf=rfc.predict_proba(data_test_in)
myFPR_train_xg,myTPR_train_xg,myTHRESHOLDS_train_xg=          roc_curve(data_train_out,
train_y_score_xg)
myFPR_test_xg,myTPR_test_xg,myTHRESHOLDS_test_xg=               roc_curve(data_test_out,
test_y_score_xg)
myAUC_train_xg = auc(myFPR_train_xg,myTPR_train_xg)
myAUC_test_xg = auc(myFPR_test_xg,myTPR_test_xg)
import matplotlib.pyplot as plt
lw = 2
plt.plot(myFPR_test_xg,myTPR_test_xg,c=mycolor1[2],label='Test ROC_curve (area = %0.2f)' %
myAUC_test_xg)
plt.plot(myFPR_train_xg,myTPR_train_xg,c=mycolor1[1],label='Train       ROC_curve       (area
= %0.2f)' % myAUC_train_xg)
plt.plot([0, 1], [0, 1], color=mycolor1[0], lw=lw, linestyle='--')
plt.xlabel('False Positive Rate',size=12)
```

```
plt.ylabel('True Positive Rate',size=12)
plt.xlim([0.0, 1.0])
plt.ylim([0.0, 1.05])
plt.legend(loc='lower right',fontsize=12)
plt.savefig('C:/Users/10699/Desktop/xlx/pic/20220220/xgb_ROC_curve',dpi=600,bbox_inches =
'tight')

#绘制混淆矩阵

def plot_confusion_matrix(cm, classes,normalize=False,cmap=plt.cm.Blues):
    """
    This function prints and plots the confusion matrix.
    Normalization can be applied by setting `normalize=True`.
    """
    if normalize:
        cm = cm.astype('float') / cm.sum(axis=1)[:, np.newaxis]
        print("Normalized confusion matrix")
    else:
        print('Confusion matrix, without normalization')
    print(cm)
    plt.imshow(cm, interpolation='nearest', cmap=cmap)
    plt.colorbar()
    tick_marks = np.arange(len(classes))
    plt.xticks(tick_marks, classes)
    plt.yticks(tick_marks, classes)
    fmt = '.2f' if normalize else 'd'
    thresh = cm.max() / 2.
    for i, j in itertools.product(range(cm.shape[0]), range(cm.shape[1])):
        plt.text(j, i, format(cm[i, j], fmt),
                 horizontalalignment="center",
                 color="white" if cm[i, j] > thresh else "black")
    plt.tight_layout()
    plt.ylabel('True label',fontsize=12)
    plt.xlabel('Predicted label',fontsize=12)

import itertools
cnf_matrix_xg = confusion_matrix(data_test_out, maxindex_xg) #计算混淆矩阵
class_names = [0,1]
plt.figure()
plot_confusion_matrix(cnf_matrix_xg, classes = class_names)   #绘制混淆矩阵
np.set_printoptions(precision=2)
xg_Accary =
[(cnf_matrix_xg[1,1]+cnf_matrix_xg[0,0])/(cnf_matrix_xg[1,1]+cnf_matrix_xg[0,1]+cnf_matrix_
xg[0,0]+cnf_matrix_xg[1,0])]
```

```
xg_Recall = [(cnf_matrix_xg[1,1]/(cnf_matrix_xg[1,1]+cnf_matrix_xg[1,0]))]
xg_Precision= [(cnf_matrix_xg[1,1]/(cnf_matrix_xg[1,1]+cnf_matrix_xg[0,1]))]
xg_Specificity= [(cnf_matrix_xg[0,0]/(cnf_matrix_xg[0,1]+cnf_matrix_xg[0,0]))]
plt.savefig('C:/Users/10699/Desktop/xlx/pic/20220220/xgb_Confusion_matrix',dpi=600,bbox_inc
hes = 'tight')

################ Logistic ################################################


from sklearn.linear_model import LogisticRegression

classifier_logistic=LogisticRegression()
classifier_logistic.fit(data_train_in,data_train_out)
data_train_pre_logistic = classifier_logistic.predict_proba(data_train_in)
data_test_pre_logistic = classifier_logistic.predict_proba(data_test_in)

train_y_score_lr = data_train_pre_logistic[:,1]
test_y_score_lr = data_test_pre_logistic[:,1]

#评价指标:AUC
plt.figure(figsize=(6,5))
myTP=0;myTN=0;FP=0;FN=0
from sklearn.metrics import roc_curve,auc,confusion_matrix
# train_y_score_rf=rfc.predict_proba(data_train_in)
# test_y_score_rf=rfc.predict_proba(data_test_in)
myFPR_train_lr,myTPR_train_lr,myTHRESHOLDS_train_lr= roc_curve(data_train_out,
train_y_score_lr)
myFPR_test_lr,myTPR_test_lr,myTHRESHOLDS_test_lr= roc_curve(data_test_out,
test_y_score_lr)
myAUC_train_lr = auc(myFPR_train_lr,myTPR_train_lr)
myAUC_test_lr = auc(myFPR_test_lr,myTPR_test_lr)
import matplotlib.pyplot as plt
lw = 2
plt.figure(figsize=(6,5))
plt.plot(myFPR_test_lr,myTPR_test_lr,c=mycolor1[2],label='Test ROC_curve (area = %0.2f)' %
myAUC_test_lr)
plt.plot(myFPR_train_lr,myTPR_train_lr,c=mycolor1[1],label='Train ROC_curve (area = %0.2f)' %
myAUC_train_lr)
plt.plot([0, 1], [0, 1], color=mycolor1[0], lw=lw, linestyle='--')
plt.xlabel('False Positive Rate',size=12)
plt.ylabel('True Positive Rate',size=12)
plt.xlim([0.0, 1.0])
plt.ylim([0.0, 1.05])
plt.legend(loc='lower right',fontsize=12)
```

```
plt.savefig('C:/Users/10699/Desktop/xlx/pic/20220220/LR_ROC_curve',dpi=600,bbox_inches = 'tight')

#绘制混淆矩阵

def plot_confusion_matrix(cm, classes,normalize=False,cmap=plt.cm.Blues):
    """
    This function prints and plots the confusion matrix.
    Normalization can be applied by setting `normalize=True`.
    """
    if normalize:
        cm = cm.astype('float') / cm.sum(axis=1)[:, np.newaxis]
        print("Normalized confusion matrix")
    else:
        print('Confusion matrix, without normalization')
    print(cm)
    plt.imshow(cm, interpolation='nearest', cmap=cmap)
    plt.colorbar()
    tick_marks = np.arange(len(classes))
    plt.xticks(tick_marks, classes)
    plt.yticks(tick_marks, classes)
    fmt = '.2f' if normalize else 'd'
    thresh = cm.max() / 2.
    for i, j in itertools.product(range(cm.shape[0]), range(cm.shape[1])):
        plt.text(j, i, format(cm[i, j], fmt),
                 horizontalalignment="center",
                 color="white" if cm[i, j] > thresh else "black")
    plt.tight_layout()
    plt.ylabel('True label',fontsize=12)
    plt.xlabel('Predicted label',fontsize=12)

maxindex_lr = np.argmax(data_test_pre_logistic,axis=1)

import itertools
cnf_matrix_lr = confusion_matrix(data_test_out, maxindex_lr) #计算混淆矩阵
class_names = [0,1]
plt.figure()
plot_confusion_matrix(cnf_matrix_lr, classes = class_names)    #绘制混淆矩阵
np.set_printoptions(precision=2)
lr_Accary = [(cnf_matrix_lr[1,1]+cnf_matrix_lr[0,0])/(cnf_matrix_lr[1,1]+cnf_matrix_lr[0,1]+cnf_matrix_lr[0,0]+cnf_matrix_lr[1,0])]
lr_Recall = [(cnf_matrix_lr[1,1]/(cnf_matrix_lr[1,1]+cnf_matrix_lr[1,0]))]
lr_Precision= [(cnf_matrix_lr[1,1]/(cnf_matrix_lr[1,1]+cnf_matrix_lr[0,1]))]
```

```
lr_Specificity= [(cnf_matrix_lr[0,0]/(cnf_matrix_lr[0,1]+cnf_matrix_lr[0,0]))]
plt.savefig('C:/Users/10699/Desktop/xlx/pic/20220220/LR_Confusion_matrix',dpi=600,bbox_inches = 'tight')
```
